# Proactive Resistance Management Studies Highlight the Role of Cytochrome P450 Genes in the Resistance of *Tuta absoluta* Against Tetraniliprole

**DOI:** 10.3390/ijms26115180

**Published:** 2025-05-28

**Authors:** Farman Ullah, Zeeshan Ullah, Hina Gul, Xiaowei Li, Yuhan Pan, Haixia Zhang, Zhijun Zhang, Jun Huang, Roditakis Emmanouil, Raul Narciso C. Guedes, Nicolas Desneux, Yaobin Lu

**Affiliations:** 1 State Key Laboratory for Quality and Safety of Agro-Products, Key Laboratory of Biotechnology in Plant Protection of MOA of China and Zhejiang Province, Institute of Plant Protection and Microbiology, Zhejiang Academy of Agricultural Sciences, Hangzhou 310021, China; farmanullah787@gmail.com (F.U.); gulhina680@gmail.com (H.G.); panyuh0306@163.com (Y.P.); zhx0422980802@163.com (H.Z.); zhijunzhanglw@hotmail.com (Z.Z.); junhuang1981@126.com (J.H.); 2 Department of Entomology, Abdul Wali Khan University Mardan, Khyber Pakhtunkhwa 23200, Pakistan; zeeshanullah212@gmail.com; 3 Department of Agriculture, School of Agricultural Sciences, Hellenic Mediterranean University, 71410 Heraklion, Greece; eroditakis@hmu.gr; 4 Departamento de Entomologia, Universidade Federal de Viçosa, Viçosa, MG 36570-900, Brazil; guedes@ufv.br; 5 Université Côte d’Azur, INRAE, CNRS, UMR ISA, 06000 Nice, France; nicolas.desneux@inrae.fr

**Keywords:** nanomaterial, RNA interference, insecticide resistance, cytochrome P450, diamide

## Abstract

The diamide insecticide tetraniliprole is a valuable tool for managing major insect pests like the invasive tomato pinworm, *Tuta absoluta* (Meyrick). However, the mechanisms underlying tetraniliprole resistance, as well as its associated fitness costs, remain unclear. In this study, we assessed the fitness of tetraniliprole-resistant (TetraRS) and susceptible (SS) strains of *T. absoluta* and conducted Illumina RNA-seq to compare their transcriptomes. We also used nanocarrier-mediated RNA interference (RNAi) to knockdown P450 genes and evaluate their role in tetraniliprole resistance. After eight generations of selection, *T. absoluta* developed a 20.80-fold resistance to tetraniliprole, accompanied by fitness costs. RNA-seq analysis revealed 3332 differentially expressed genes (DEGs), with 1707 upregulated and 1625 downregulated in the TetraRS compared to the SS strain. Gene Ontology (GO) annotations showed significant enrichment in categories related to metabolic processes, cellular processes, catalytic activity, cellular anatomical entity, and binding. These genes were also identified in key KEGG pathways such as cytochrome P450, drug metabolism, carbon metabolism, oxidative phosphorylation, fatty acid metabolism, and protein processing. RT-qPCR analysis confirmed that P450 genes (*CYP405D1*, *CYP6AB269*, and *CYP4AU1*) were upregulated in TetraRS insects, in line with the RNA-seq results. Cytochrome P450 activity was significantly higher in the TetraRS strain than in the SS strain. Notably, nano-encapsulated dsRNA targeting these overexpressed P450 genes increased the susceptibility of *T. absoluta* to tetraniliprole. Further, cytochrome P450 activity was significantly reduced following silencing of P450 genes. These findings suggest that multiple genes and pathways, particularly P450 genes, contribute to tetraniliprole resistance in *T. absoluta*. This study provides valuable insights into the molecular mechanisms underlying insecticide resistance in this key pest species.

## 1. Introduction

Insecticidal molecules, though controversial, remain crucial tools for pest management against several arthropod pest species. Despite increasing calls for greener agriculture practices, non-target effects, and ongoing regulatory restrictions [1,2], chemical insecticides are still widely used for their effectiveness in providing immediate, cost-efficient pest control. This is especially important when dealing with invasive species in newly invaded areas [3,4,5,6]. One such invasive pest species is the South American tomato pinworm, *Tuta absoluta* (Meyrick) (Lepidoptera: Gelechiidae) [4,7].

The tomato pinworm, which originated from the central Peruvian highlands [4], invaded Europe in 2006 and has now spread to over 90 countries, including China [5]. This oligophagous pest primarily attacks Solanaceae crops such as tomatoes, potatoes, eggplants, tobacco, etc. By mining and feeding on leaves, buds, stems, and fruits during its larval stage, *T. absoluta* can cause 80–100% crop loss if not managed properly and promptly, depending on the infestation level [5]. Its rapid spread and high feeding efficiency are driven by several critical biological and ecological characteristics that favor its expansion, posing a serious threat to agricultural production worldwide [5,6].

Tetraniliprole, a recently introduced diamide insecticide, and other compounds of the same group show promising efficacy against several agricultural pests, including the tomato pinworm. These insecticides target the ryanodine receptor, which regulates calcium ion balance in insect muscle cells. Disruption of this receptor leads to gut toxicity, paralysis, muscle contractions, and eventual death [8]. Tetraniliprole is especially effective against larvae, making it a valuable tool in integrated pest management (IPM) programs to control not only major lepidopteran pests [9,10,11] but also other insect pests [12,13].

However, the frequent use of diamides has led to increasing insecticide resistance [14,15]. Recently, a strain of *T. absoluta* was reported to exhibit a 36.2-fold resistance to tetraniliprole, along with 12.2- and 6.7-fold cross-resistance to chlorantraniliprole and flubendiamide, respectively [16]. While resistance to tetraniliprole has not been widely reported, laboratory selection often results in resistance development in lepidopteran pests [17,18,19]. For instance, *T. absoluta* strains have developed resistance to diamides due to mutations in the ryanodine receptor [20]. Overexpression of P450 detoxification enzymes has also been linked to resistance in *T. absoluta* when exposed to abamectin and chlorantraniliprole. However, the specific role of P450 genes in tetraniliprole resistance in *T. absoluta* remains unclear.

Understanding the molecular mechanisms of tetraniliprole resistance, particularly by targeting cytochrome P450 genes via nanocarrier-mediated RNA interference (RNAi), is crucial for developing effective resistance monitoring strategies. RNAi is a post-transcriptional gene silencing technique that uses artificial RNA molecules to silence specific genes [21]. Over the past two decades, RNAi has been established as a powerful tool for gene silencing, used both in functional validation studies of resistance-related genes and directly in the development of biotechnological products [22,23]. The delivery mechanism is crucial for the success of RNAi experiments [23]. Successful RNAi experiments depend heavily on the delivery mechanism. In this context, nanotechnology plays a key role by enabling the efficient delivery of RNAi molecules, thereby enhancing the applicability and effectiveness of RNAi-based approaches for sustainable pest control [24]. Nanocarriers, small delivery systems for RNA molecules, have been widely used to enhance RNAi stability, targeted delivery, and cellular uptake in various insect species [24,25]. Star polycation (SPc), a branched polymer with multiple positively charged arms, has been developed as a cost-effective gene vector. It spontaneously assembles with dsRNA through electrostatic interactions, ultimately protecting dsRNA from degradation by RNase A [26,27]. The stability of nanocarrier-loaded dsRNA significantly increases the likelihood of dsRNA binding to insect cells, thereby enhancing the efficiency of RNAi [28,29]. Recently, it was reported that the successful downregulation of *Takr-h1* in *T. absoluta* after feeding *dskr-h1*/SPc nanoparticle complexes for 24 h [30].

In the current study, we conducted Illumina RNA-seq to compare the transcriptomes of tetraniliprole-resistant (TetraRS) and susceptible (SS) strains of *T. absoluta*, aiming to identify key genes and pathways associated with tetraniliprole resistance. Three cytochrome P450 genes (*CYP405D1*, *CYP6AB269*, and *CYP4AU1*) were selected based on their overexpression for nanocarrier-mediated RNAi experiments to assess their role in tetraniliprole resistance. The results provide a foundation for further exploration of the molecular mechanisms underlying tetraniliprole resistance in *T. absoluta*.

## 2. Results

### 2.1. Tetraniliprole Relative Toxicity and Resistance Development

Bioassays were conducted on the susceptible strain of *T. absoluta* to assess the toxicity of tetraniliprole. The toxicity results indicated that the LC_50_ of tetraniliprole against the SS *T. absoluta* was 0.03 mg L^−1^ (0.02–0.03 mg L^−1^). The tetraniliprole-resistant strain (TetraRS) of *T. absoluta* was established following successive tetraniliprole selection for eight generations under laboratory conditions (Table 1). The LC_50_ values of tetraniliprole increased in the subsequent generations due to continuous selection pressure (F1–F8). In the first three generations, the LC_50_ values gradually increased from 0.03 mg L^−1^ to 0.08 mg L^−1^ with 1.03- to 2.65-fold tetraniliprole resistance compared to the susceptible strain of *T. absoluta* (Table 2). However, in F4 to F8 generations of *T. absoluta*, the LC_50_ values were sharply increased to 0.15, 0.25, 0.36, 0.48, and 0.60 mg L^−1^, respectively. Following successive selection for eight generations, *T. absoluta* developed 20.80-fold resistance against tetraniliprole as compared to the susceptible strain (Table 1).

### 2.2. Effects of Tetraniliprole Resistance on Life-History Traits and P450 Enzyme Activity

**Figure 1:**
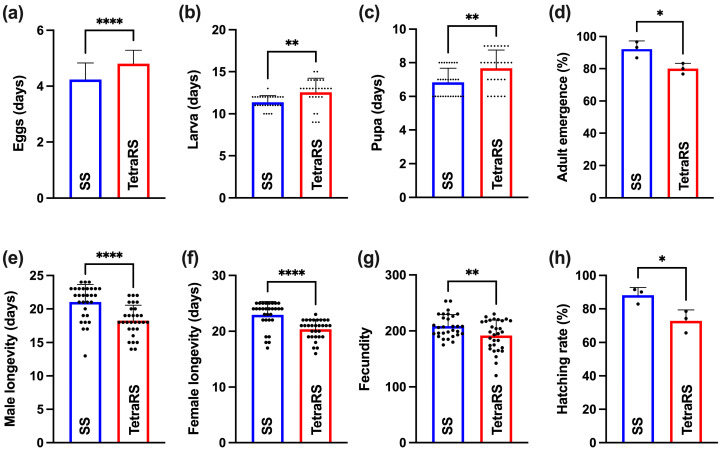
Life-history traits of tetraniliprole-resistant (TetraRS) and susceptible (SS) strains of *Tuta absoluta*. Sample sizes: (**a**) Sixty-three and sixty-five eggs were used for SS and TetraRS groups, respectively; (**b**) twenty-nine larvae were used for each SS and TetraRS group; (**c**) thirty pupae were used for each SS and TetraRS cohort; (**d**) three replicates were used for each group (SS and TetraRS), and thirty individuals were used for each replicate; (**e**) thirty male *T. absoluta* were used for each cohort (SS and TetraRS); (**f**) thirty female individuals were used for each SS and TetraRS group; (**g**) thirty pairs of male and female *T. absoluta* were used for each cohort, i.e., SS and TetraRS; (**h**) three replicates were used for each group, and seventy eggs were used for each replicate. Data presented as mean ± SE, and the asterisks *, **, and **** show significant differences at *p* < 0.05, *p* < 0.001, and *p* < 0.0001 based on Student’s *t*-test.

The influence of tetraniliprole resistance on the life-history traits of *T. absoluta* is shown in Figure 1. Compared to the SS strain, the egg incubation time (*t*-test: *t* = 5.61, df = 116, *p* < 0.001) and the larval (*t*-test: *t* = 3.41, df = 56, *p* = 0.001) and pupal development times (*t*-test: *t* = 3.32, df = 58, *p* = 0.002) were significantly prolonged in the TetraRS strain. However, both male (*t*-test: *t* = 4.37, df = 58, *p* < 0.001) and female longevity (*t*-test: *t* = 4.76, df = 58, *p* < 0.001) were significantly reduced in TetraRS. Additionally, fecundity (*t*-test: *t* = 2.68, df = 58, *p* = 0.01), hatching rate (t-test: *t* = 3.30, df = 4, *p* = 0.03), and adult emergence (*t*-test: *t* = 3.48, df = 4, *p* = 0.03) were significantly lower in TetraRS compared to the SS strain (Figure 1).

**Figure 2:**
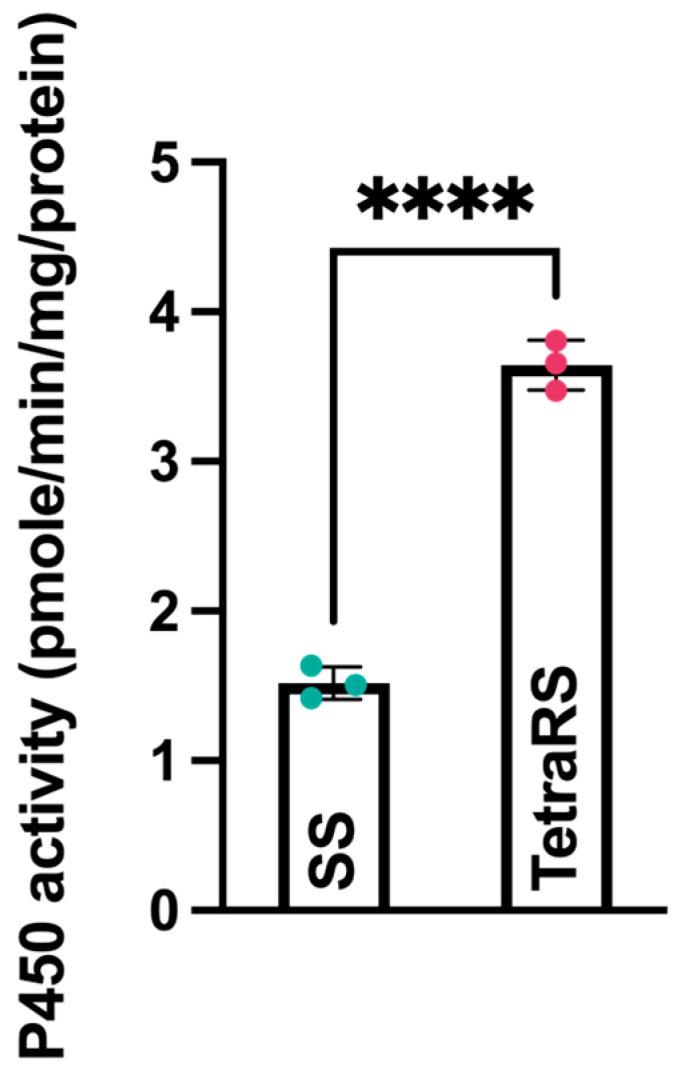
P450 enzyme activity in tetraniliprole-resistant (TetraRS) and susceptible (SS) strains of *Tuta absoluta*. Data presented as mean ± SE of the three independent biological replicates. The asterisks **** represent significant differences at *p* < 0.0001, based on Student’s *t*-test.

Cytochrome P450 enzyme activity was markedly elevated in the TetraRS strain compared to the SS strain (*t*-test: *t* = −18.51, df = 4, *p* < 0.001), suggesting a strong correlation between P450 overexpression and tetraniliprole resistance (Figure 2).

### 2.3. Transcriptome Data Annotation

Transcriptomic analysis showed that clean reads for TetraRS ranged from 23,393,925 to 25,200,234 and for SS from 23,470,720 to 26,083,946 (Table 2). Clean base counts ranged from 6,999,448,021 to 7,540,917,370 in TetraRS and from 7,024,623,252 to 7,806,153,973 in SS. The GC content ranged from 46.28 to 47.11% in TetraRS and 45.92 to 47.76% in SS. Quality scores (Q20 and Q30) were high for both strains, with values ranging from 98.07 to 98.25% for Q20 and 94.84 to 95.35% for Q30.

**Table 2:** Sequencing data of susceptible (SS) and tetraniliprole-resistant (TetraRS) strains of *Tuta absoluta*.

Samples	Reads	Bases	GC (%)	N (%)	Q20 (%)	Q30 (%)
SS-1	23,470,720	7,024,623,252	46.75	0.04	98.24	95.34
SS-2	24,722,615	7,400,591,111	45.92	0.04	98.07	94.84
SS-3	26,083,946	7,806,153,973	46.58	0.04	98.25	95.35
TetraRS-1	23,478,474	7,027,641,397	46.28	0.04	98.13	94.99
TetraRS-2	25,200,234	7,540,917,370	46.7	0.04	98.2	95.24
TetraRS-3	23,393,925	6,999,448,021	47.11	0.04	98.14	95.06

### 2.4. Differentially Expressed Genes in TetraRS and SS Tuta Absoluta

**Figure 3:**
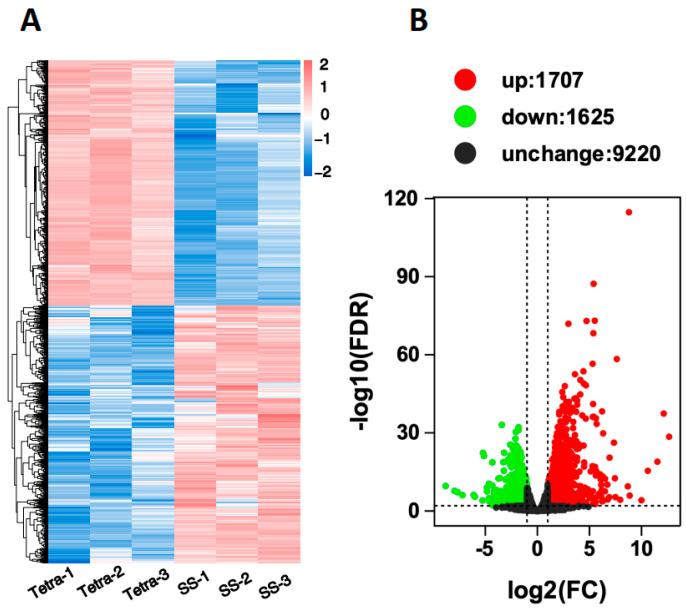
RNA-seq data of tetraniliprole-resistant (TetraRS) and susceptible strains (SS) of *Tuta absoluta*. (**A**) In the heatmap, the expression level of genes (FPKM) was normalized by log10, i.e., log10 (FPKM + 0.000001), and presented as different colors based on the scale bar. (**B**) In the volcano plot, each dot represents a gene. The *x*-axis shows the log2Fold expression change, and the *y*-axis indicates the −log10 (FDR).

A hierarchical clustering analysis grouped differentially expressed genes (DEGs) based on expression patterns, indicating consistent expression levels among replicates (Figure 3A). A total of 3332 DEGs were identified between the TetraRS and SS strains, with 1707 genes (51.23%) upregulated and 1625 genes (48.56%) downregulated in TetraRS compared to SS. The volcano plot highlighted significant up- and down-regulation of various DEGs between the two strains (Figure 3B).

### 2.5. Functional Annotation of DEGs

**Figure 4:**
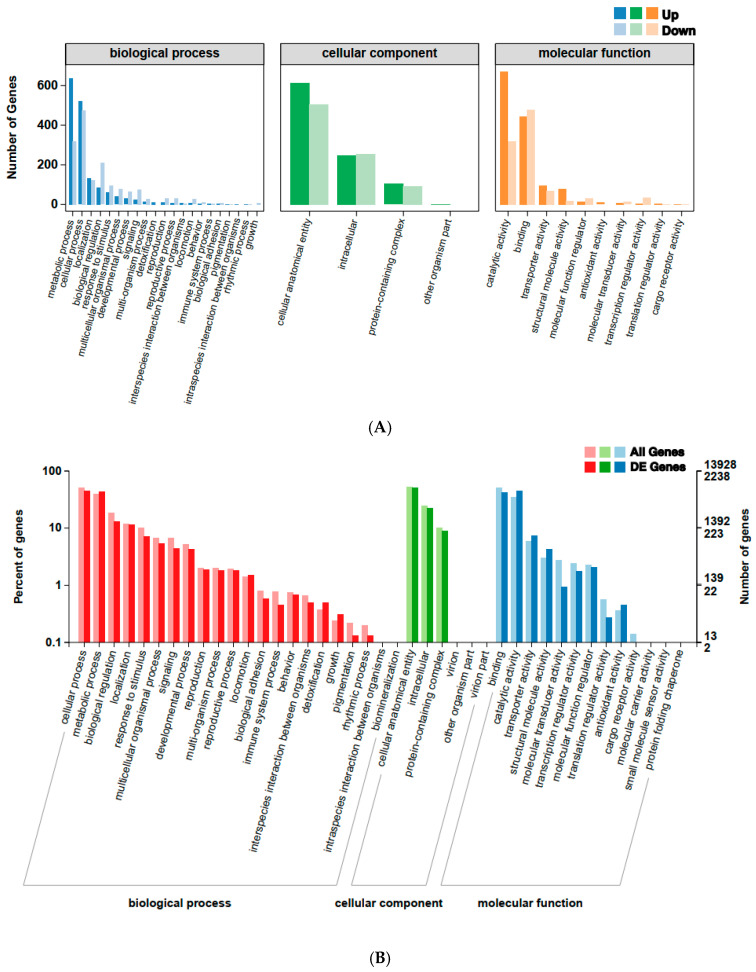
Gene Ontology (GO) enriched terms of the up- and down-regulated genes of tetraniliprole-resistant (TetraRS) and susceptible strains (SS) of *Tuta absoluta* (**A**). The *x*-axis showed GO terms and classifications, while the *y*-axis indicated the number of DEGs annotated to the term and the percentage of all DEGs. Gene Ontology (GO) enriched terms of the total number of genes and differentially expressed genes (DEGs) in tetraniliprole-resistant (TetraRS) and susceptible strains (SS) of *Tuta absoluta* (**B**). The *x*-axis shows GO terms and classifications, the left *y*-axis indicates the percentage, and the right *y*-axis shows the number of all genes and DEGs.

**Figure 5:**
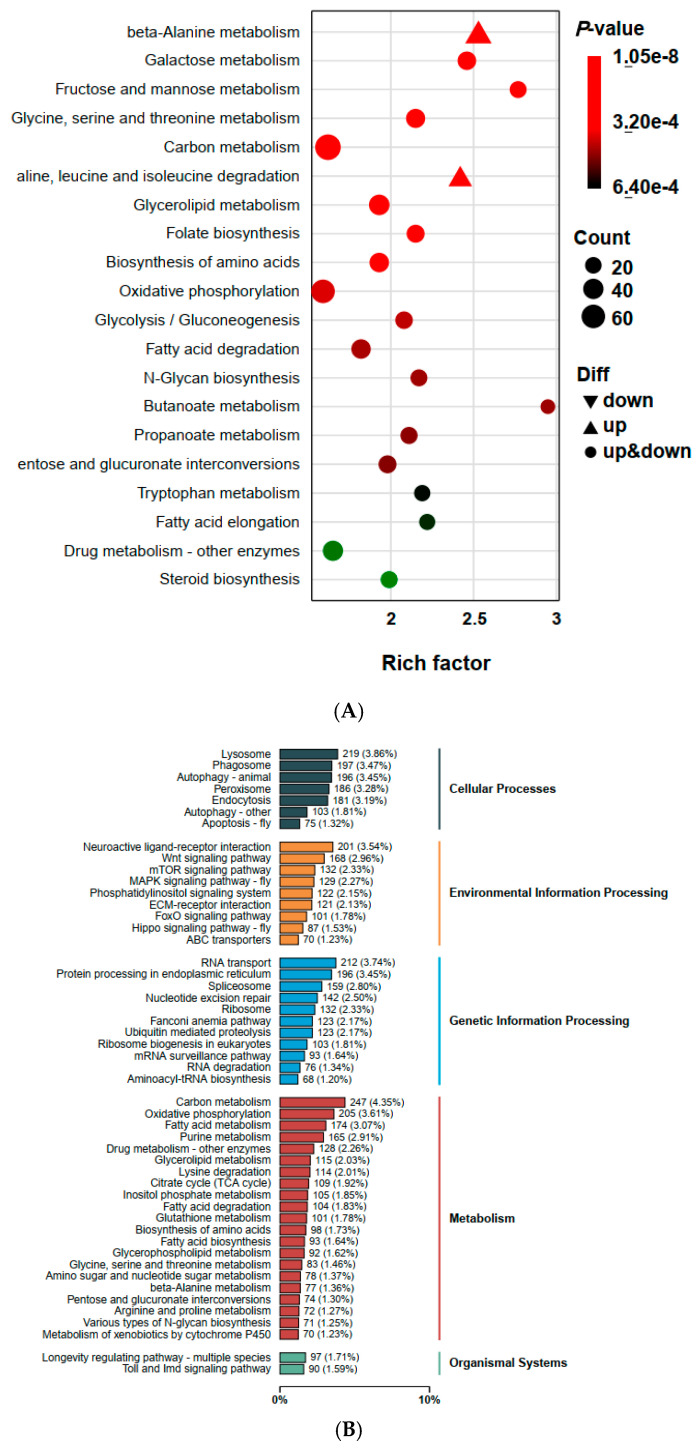
KEGG pathway enrichment on DEGs of tetraniliprole-resistant (TetraRS) and susceptible (SS) strains of *Tuta absoluta* (**A**). Each dot represents a KEGG pathway. *y*-axis: Pathway; *x*-axis: Rich factor. Enrichment factor is calculated as “Enrichment factor = (Ratio of DEGs annotated to the term over all DEGs)/(Ratio of genes annotated to the term over all genes). The KEGG annotations of DEGs classification based on the type of pathways (**B**). *y*-axis: KEGG pathway terms; *x*-axis: number and percentage of genes annotated to the KEGG pathway.

Gene Ontology (GO) analysis indicated that in the biological process category, genes were enriched for cellular processes, metabolic processes, and biological regulation. In the molecular function category, genes associated with catalytic activity and binding were predominant. The cellular component category showed significant enrichment for genes involved in cellular anatomical entities and intracellular processes (Figure 4). KEGG pathway analysis identified 1001 DEGs associated with metabolism, making it the most dominant pathway category. Other enriched pathways included cellular processes, genetic information processing, environmental information processing, and organismal systems. Key metabolic pathways included carbon metabolism, oxidative phosphorylation, and glycerolipid metabolism. The cellular process pathways featured lysosomes and peroxisomes, while protein processing in the endoplasmic reticulum and neuroactive ligand-receptor interaction were notably enriched in other categories (Figure 5).

### 2.6. Expression Levels of Candidate Genes

**Figure 6:**
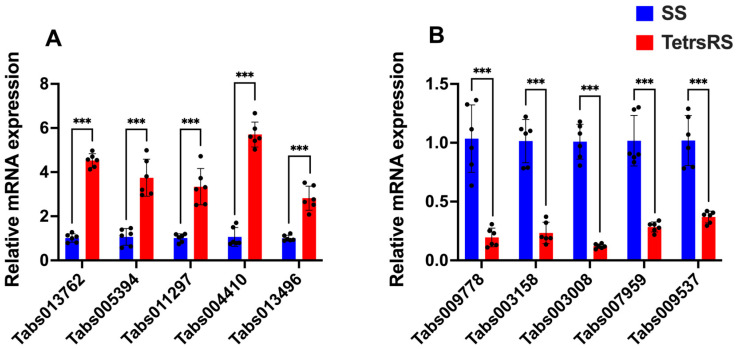
RT-qPCR validation of the upregulated (**A**) and downregulated (**B**) genes in tetraniliprole-resistant (TetraRS) and susceptible (SS) strains of *Tuta absoluta*. Data presented as mean ± SE of the three independent biological replicates. The asterisks *** represent significant differences at *p* < 0.001, based on Student’s *t*-test.

To confirm the transcriptomic data, RT-qPCR was performed on the selected upregulated (Tabs013762, Tabs005394, Tabs011297, Tabs004410, and Tabs013496) and downregulated (Tabs009778, Tabs003158, Tabs003008, Tabs007959, and Tabs009537) genes in the SS and TetraRS strains of *T. absoluta* (Figure 6). RT-qPCR results of upregulated and downregulated genes were consistent with the RNA-seq data, validating the accuracy of the transcriptomic analysis. The expression levels of Tabs013762 (*t* = −16.095, *df* = 10, *p* < 0.001), Tabs005394 (*t* = −23.720, *df* = 10, *p* < 0.001), Tabs011297 (*t* = −7.130, *df* = 10, *p* < 0.001), Tabs004410 (*t* = −6.750, *df* = 10, *p* < 0.001), and Tabs013496 (*t* = −7.900, *df* = 10, *p* < 0.001) were significantly increased by 5.70-, 4.52-, 3.74-, 3.33-, and 2.82-fold, respectively, as compared to SS *T. absoluta* (Figure 6A). The expression levels of Tabs009778 (*t* = 14.577, *df* = 10, *p* < 0.001), Tabs003158 (*t* = 6.915, *df* = 10, *p* < 0.001), Tabs003008 (*t* = 9.338, *df* = 10, *p* < 0.001), Tabs007959 (*t* = 8.216, *df* = 10, *p* < 0.001), and Tabs009537 (*t* = 7.288, *df* = 10, *p* < 0.001) were substantially decreased by 0.12-, 0.19-, 0.23-, 0.28-, and 0.36-fold in TetraRS *T. absoluta* as compared to the SS group (Figure 6B).

### 2.7. Nanocarrier-Mediated Silencing of P450 Genes in Resistant Tuta absoluta

**Figure 7:**
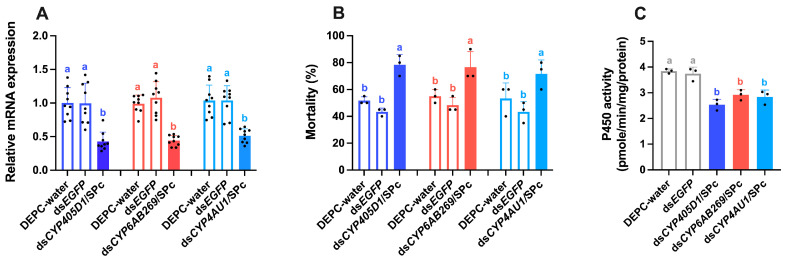
Nanocarrier-mediated RNAi of *CYP405D1*, *CYP6AB269*, and *CYP4AU1* increases the tetraniliprole susceptibility of tetraniliprole-resistant strains (TetraRS) of *Tuta absoluta*. (**A**) The expression levels of P450 genes after feeding of TetraRS strain of *T. absoluta* on ds*CYP405D1*/SPc, ds*CYP6AB269*/SPc, ds*CYP4AU1*/SPc, ds*EGFP*/SPc, and DEPC water. Data presented as mean ± SE of the three independent biological replicates. (**B**) Mortality rates (%) and (**C**) P450 enzyme activity of TetraRS *T. absoluta* following 48 h of feeding on ds*CYP405D1*/SPc, ds*CYP6AB269*/SPc, ds*CYP4AU1*/SPc, ds*EGFP*/SPc, and DEPC water after treatment with 0.603 mg/L of tetraniliprole (LC_50_ value of tetraniliprole to TetraRS). Letters above the bars represent significant differences at *p* < 0.05 level using one-way analysis of variance with Tukey’s HSD test (IBM, SPSS Statistics, version 29, Armonk, NY, USA).

Several P450 genes, such as *CYP405D1*, *CYP6AB269*, and *CYP4AU1,* were knocked down to validate their role in tetraniliprole resistance in *T. absoluta*. Following feeding on dsRNA/nanoparticle complexes for 48 h, RT-qPCR revealed significant knockdown of the target genes: *CYP405D1* (*F*_2,26_ = 18.237, *p* < 0.001, 0.42-fold), *CYP6AB269* (*F*_2,26_ = 40.236, *p* < 0.001, 0.44-fold), and *CYP4AU1* (*F*_2,26_ = 22.410, *p* < 0.001, 0.51-fold) compared to controls (Figure 7A). Further, feeding the dsRNA-treated TetraRS larvae on tetraniliprole-treated leaves (LC_50_ = 0.603 mg/L) significantly increased mortality rates, with 78.33% (*F*_2,8_ = 40.111, *p* < 0.001), 76.66% (*F*_2,8_ = 10.304, *p* = 0.011), and 71.66% (*F*_2,8_ = 6.194, *p* = 0.035) mortality observed for ds*CYP405D1*/SPc, ds*CYP6AB269*/SPc, and ds*CYP4AU1*/SPc treatments, respectively, compared to ds*EGFP*/SPc and DEPC water (Figure 7B). The cytochrome P450 enzyme activity was significantly reduced (*F*_5,17_ = 21.067, *p* < 0.001) in ds*CYP405D1*/SPc, ds*CYP6AB269*/SPc, and ds*CYP4AU1*/SPc treatments compared to ds*EGFP*/SPc and DEPC water (Figure 7C).

## 3. Discussion

The South American tomato pinworm, *T. absoluta*, is one of the most destructive invasive pests damaging tomatoes worldwide, including China [6,31]. While there are several alternative techniques to control this key pest, chemical applications remain a pivotal management tool across the globe [5]. However, the broad and often misapplication of insecticides against *T. absoluta* has accelerated the development of resistance. The pest’s global invasion highlights its remarkable ability to develop resistance to commonly used insecticides [3]. Resistance to several major insecticide classes, including diamides, spinosyns, oxadiazines, pyrethrins, and organophosphates, has been reported, coinciding with the pest’s rapid global spread [4,7].

In our study, *T. absoluta* developed 20.80-fold resistance after exposure to tetraniliprole for over eight continuous generations. We also observed resistance-associated fitness costs, reflected in extended developmental time, lower adult longevity, and reduced fecundity, hatching, and adult emergence in the resistant insects. According to life-history theory, trade-offs arise when two or more traits compete for the same resources, requiring organisms to balance energetically costly traits such as reproduction, growth, and longevity [32]. The reduced longevity and fecundity in resistant *T. absoluta* may result from the energy trade-off between adaptation and life-history traits when dealing with insecticide stress [33]. Resistance-induced fitness costs have been widely reported in insects exposed to various insecticides [34].

The fast evolution of insecticide resistance in pests like *T. absoluta* is driven by various mechanisms that need further investigation. Amplification, upregulation, and/or modification of genes encoding microsomal oxidases, such as cytochromes P450 (CYPs), glutathione S-transferases (GSTs), and carboxylesterases (CarEs), are common mechanisms of metabolic resistance [35]. In addition to metabolic resistance, insects may employ target-site, penetration, and behavioral resistance mechanisms [36,37]. Therefore, in-depth research on genes and pathways linked to insecticide resistance is crucial to developing effective pest management strategies for this key pest.

Illumina sequencing technology provides a powerful tool for generating comprehensive transcriptomic data, enabling in-depth exploration of insecticide resistance mechanisms [38,39]. Building on such transcriptomic insights, RNA interference (RNAi) has emerged as a valuable tool for functional validation of resistance-related genes. In recent years, nano-encapsulated dsRNA delivery systems have been extensively employed to enhance dsRNA’s stability and targeted delivery, further advancing the application of RNAi in pest management strategies. This nanocarrier-mediated technique has shown significant potential in enhancing the stability and efficiency of RNAi in both in vitro and in vivo applications. In this study, we used high-throughput transcriptome analysis on tetraniliprole-resistant (TetraRS) and susceptible (SS) strains of *T. absoluta* to identify key resistance-related genes and pathways. Furthermore, cytochrome P450 genes were functionally studied in the TetraRS strain via a nanocarrier-mediated RNA interference (RNAi) approach.

Insects exposed to natural and synthetic xenobiotics, including insecticides, develop various adaptive strategies, such as target-site resistance, metabolic resistance, and penetration resistance, as well as an emerging new mode of resistance, i.e., sequestration resistance, to break down these substances [40]. Differentially expressed genes (DEGs) and related pathways provide key insights into the insect’s response to stress [41]. We investigated the molecular mechanisms underlying tetraniliprole resistance in the TetraRS strain of *T. absoluta* by analyzing DEGs and associated metabolic pathways. RNA-seq results revealed 3332 DEGs (1707 upregulated and 1625 downregulated) between TetraRS and SS individuals. Gene ontology (GO) annotation indicated that these genes were highly enriched in terms such as cellular processes, metabolic processes, biological regulation, catalytic activity, and binding. The KEGG pathway analysis showed significant enrichment in pathways including carbon metabolism, oxidative phosphorylation, glycerolipid metabolism, lysosome, peroxisome, protein processing in the endoplasmic reticulum, and neuroactive ligand-receptor interaction, suggesting their key roles in tetraniliprole resistance.

The GO and KEGG analyses revealed several genes associated with detoxification enzymes. Organisms activate various metabolic activities to increase their ability to withstand external stress [42]. For example, when *T. absoluta* was treated with abamectin and chlorantraniliprole, metabolic pathways related to detoxification were highly enriched [43]. The fatty acid metabolism, drug metabolism, glutathione metabolism, pyrimidine metabolism, and cytochrome P450 pathways were enriched in *T. absoluta* exposed to the abamectin/chlorantraniliprole complex [43], indicating adaptive strategies to counter stress.

Cytochrome P450 enzymes play a critical role in metabolizing xenobiotics and detoxifying pesticides [44]. Overexpression of the P450 monooxygenase enzyme is a primary mechanism of insecticide resistance in several insects [45,46]. In our RNA-seq study, three P450 genes—*CYP405D1*, *CYP6AB269*, and *CYP4AU1*—were overexpressed in TetraRS; validated by RT-qPCR. Moreover, cytochrome P450 enzyme activity was significantly higher in TetraRS compared to SS *T. absoluta*. These results suggest that these P450 genes are linked to tetraniliprole resistance in *T. absoluta*. Previous studies have reported the involvement of P450 genes in resistance to other insecticides in *T. absoluta* [43,47]. To better understand the role of P450 genes in tetraniliprole resistance, we used nanomaterial-encapsulated dsRNA to silence *CYP405D1*, *CYP6AB269*, and *CYP4AU1*. The RNAi silencing significantly enhanced the susceptibility of TetraRS to tetraniliprole as well as reduced the cytochrome P450 enzyme activity, further confirming that these genes are crucial for resistance. Similar findings have been reported in other lepidopteran pests, including *Spodoptera* sp. [48] and *Plutella xylostella* [49], and non-lepidopteran species like *Aphis gossypii* [50] and *Laodelphax striatellus* [51]. Laboratory selection experiments highlighted the role of P450 detoxification in the resistance of *T. absoluta* to tetraniliprole, which resulted in moderate resistance levels in just eight selection cycles. However, tetraniliprole selection was conducted on a susceptible strain of *T. absoluta* that did not possess target site RYR mutations associated with diamide resistance. In addition, in this study, the overexpression of P450 genes was associated with significant fitness cost, potentially restricting the long-term involvement of the particular detoxification mechanism in ‘future’ field-evolved resistance. Although there is significant scientific evidence supporting proactive resistance management of the pest, the role of the particular P450 genes remains to be confirmed in planta. Further, the current study provides a foundation for several promising future directions in pest management. For instance, developing P450-based diagnostic tools could enable early detection of resistance in pest populations, allowing for timely and targeted interventions. By identifying specific P450 gene variants linked to resistance, we can create assays to monitor resistance levels in the field. Furthermore, implementing a multifaceted approach that combines cultural practices, insecticide rotation, and RNA interference (RNAi) techniques could reduce the selection pressure on Tuta absoluta populations. Additionally, nanocarrier-mediated RNAi could be particularly effective by targeting key lethal genes, thereby enhancing pest control while minimizing environmental impact.

In summary, the current study showed tetraniliprole resistance (20.80-fold) along with fitness costs in *T. absoluta* following continuous selection over eight generations. Further, we employed RNA-seq and nanocarrier-mediated RNAi to investigate the functional role of several cytochrome P450 genes in tetraniliprole resistance in *T. absoluta*. The findings provide important insights into the molecular mechanisms underlying resistance to diamide insecticides in this invasive pest species, highlighting the role of P450 genes and their potential as targets for future pest management strategies. However, fitness costs observed in laboratory settings may not directly translate to field contexts. While the controlled environment of the lab facilitates the observation of specific genetic and physiological responses, the complexities and variability of natural habitats can significantly influence these dynamics. Consequently, additional field studies are essential to validate the ecological relevance of the current laboratory findings and to understand their implications for effective pest management strategies.

## 4. Materials and Methods

### 4.1. Insect Rearing

The *T. absoluta* population was initially collected from a tomato field in Yuxi, Yunnan, China. The susceptible strain (SS) of *T. absoluta* was established under laboratory conditions following continuous rearing over several generations without any insecticide exposure. Fresh tomato plants (insecticide-free) were used to maintain the population in the laboratory (25 ± 1 °C, a 16L:8D photoperiod, and 60 ± 5% relative humidity).

### 4.2. Bioassays

To standardize the age and developmental stage of all *T. absoluta* larvae, adult moths were allowed to lay eggs on tomato plants for 12 h. The plants were then moved to clean cages. The toxicity of tetraniliprole against 3rd instar *T. absoluta* larvae was evaluated using the leaf-dip bioassay method recommended by the Insecticide Resistance Action Committee (IRAC). In brief, following preliminary bioassays, 0.0078, 0.0156, 0.0312, 0.0625, 0.125, 0.25, and 0.5 mg/L concentrations of tetraniliprole were prepared in deionized water. Fresh, untreated tomato leaves were dipped in the prepared concentrations for 15 s, air-dried at room temperature, and placed in Petri dishes lined with filter paper. The deionized water was used as a control in all bioassays. The petiole of each leaf was wrapped with cotton wool to maintain moisture. For each tetraniliprole concentration and the control, three replicates were performed, with 20 second-instar larvae in each replicate. The bioassays were conducted under laboratory conditions at 25 ± 1 °C, 60 ± 5% RH, and a 16:8 light/dark photoperiod. Mortality was assessed after 48 h, with larvae considered dead if they did not respond to gentle prodding with a brush.

### 4.3. Resistance Selection

The tetraniliprole-resistant strain (TetraRS) of *T. absoluta* was developed from the susceptible strain (SS) after eight generations of tetraniliprole selection. The tetraniliprole toxicity to *T. absoluta* was checked at each generation by conducting bioassays as mentioned in Section 4.2. Seven concentrations were used, and each concentration included three replicates. Twenty *T. absoluta* larvae were used per replicate. Based on the parental generation bioassay results, the tetraniliprole concentrations were gradually increased for the subsequent generation selection. The selection pressure during the experiment was maintained at 60–80% mortality of *T. absoluta*. The susceptible strain (SS) of *T. absoluta* was reared separately using insecticide-free tomato plants as discussed in Section 4.1. All bioassays and selection experiments were conducted under laboratory conditions (25 ± 1 °C, 60 ± 5% RH, and a 16:8 light/dark photoperiod).

### 4.4. Fitness Comparisons

To evaluate the impact of tetraniliprole resistance on insect fitness, developmental duration, adult longevity, fecundity, hatching rate, and adult emergence were recorded for both the SS and TetraRS strains. Sixty-three and fifty-five eggs from the SS and TetraRS strains, respectively, were transferred to Petri dishes with fresh tomato leaves. The egg incubation period and hatching rate were recorded daily. Twenty-nine newly hatched larvae from each strain were placed in fresh Petri dishes containing tomato leaves. The petiole of each leaf was covered to retain moisture. The larval developmental period was monitored daily. Upon pupation, 30 individuals from each strain were transferred to glass tubes (1.5 cm diameter, 8 cm height), and the pupal developmental period and adult emergence were documented. Thirty pairs of newly emerged adult males and females from both strains were placed in glass tubes (3.0 cm diameter, 20 cm height), where they were provided with fresh tomato leaves and a 10% honey solution. The longevity of the adults and fecundity were recorded daily. All fitness experiments were performed under the same laboratory conditions mentioned above.

### 4.5. P450 Enzyme Activity

The CYP450 enzyme activity in both SS and TetraRS strains of *T. absoluta* was assessed using an insect CYP450 ELISA Kit No. JL22832 (Shanghai Jianglai Industrial Limited By Share Ltd., Shanghai, China), following the manufacturer’s protocol. In brief, 50 μL of each sample was added to the wells of a microtiter plate, followed by 100 μL of enzyme conjugate, except in the blank wells. The plate was covered and incubated at 37 °C for 60 min, followed by four washing cycles. Subsequently, 50 μL of carbamide peroxide (substrate A) and 50 μL of TMB (3,3′,5,5′-Tetramethylbenzidine) reagent (substrate B) were added to each well, and the plate was gently mixed and incubated at 37 °C for 15 min. After adding 50 μL of stop solution, the optical density (O.D.) was immediately measured at 450 nm using a microtiter plate reader.

### 4.6. RNA Extraction and Transcriptome Sequencing

The total RNA was extracted from the 3rd instar *T. absoluta* of tetraniliprole-resistant (TetraRS) and susceptible strains (SS) using TRlzol Reagent (Life Technologies, Carlsbad, CA, USA). Five larvae were pooled as one biological replicate, and three replicates were used for each group. The concentration and purity of the RNA were checked with a NanoDrop 2000 spectrophotometer (Thermo Fisher Scientific, Wilmington, DE, USA). RNA integrity was evaluated using the RNA Nano 6000 Assay Kit on an Agilent Bioanalyzer 2100 system (Agilent Technologies, Santa Clara, CA, USA). All sequencing libraries were prepared using the NEBNext Ultra™ RNA Library Prep Kit for Illumina (NEB, 240 County Road, Ipswich, MA, USA) following the recommended protocol. The index-coded samples were clustered using a cBot Cluster Generation System with TruSeq PE Cluster Kit v4-cBot-HS (Illumina, San Diego, CA, USA), following the manufacturer’s instructions. Once the clusters were generated, the library preparations were sequenced on an Illumina platform, resulting in paired-end reads. The clean reads were mapped with the reference genome using Hisat2. The gene function was annotated based on the non-redundant (Nr) protein and non-redundant nucleotide (Nt) database (http://www.ncbi.nlm.nih.gov/, accessed on 25 April 2024), Gene Ontology (GO) (http://www.geneontology.org, accessed on 25 April 2024), SwissProt (http://www.expasy.ch/sprot/, accessed on 25 April 2024), Kyoto Encyclopedia of Genes and Genomes (KEGG) (http://www.genome.jp/kegg/, accessed on 25 April 2024), eggNOG (http://eggnogdb.embl.de/, accessed on 25 April 2024), Pfam (Protein family), and KOG/COG (Clusters of Orthologous Groups of proteins). For the differential expression analysis, DESeq2 was used to determine the expression levels of unigenes. The resulting *p*-values were adjusted using Benjamini and Hochberg’s approach for controlling the false discovery rate. The differentially expressed unigenes were selected with log2 (fold change) > 1 or log2 (fold change) < −1 and statistical significance (*p* < 0.05) using the R package edgeR 3.32.1.

### 4.7. RT-qPCR of Targeted Genes

Several upregulated and downregulated genes were selected from the transcriptome data for validation via quantitative real-time PCR (RT-qPCR). Total RNA was extracted from 3rd instar larvae of the SS and TetraRS strains using the RNAsimple Total RNA kit (Tiangen Biotechnology, Beijing, China). Five larvae were pooled as one biological replicate. RNA quality was confirmed using the Agilent 2100 Bioanalyzer. cDNA was synthesized from 1 μg of total RNA using the iScript cDNA Synthesis Kit (Bio-Rad, Hercules CA, USA). RT-qPCR was performed on a CFX Connect Real-Time System (Bio-Rad, Hercules CA, USA), with reactions containing 5 μL of 2× Kappa SYBR Green qPCR mix, 0.2 μL of forward and reverse primers (10 μM), 1 μL of cDNA template, and nuclease-free water in a final volume of 10 μL. Cycling conditions included an initial denaturation at 95 °C for 45 s, followed by 40 cycles of 95 °C for 15 s, 50–65 °C for 15 s, and 70 °C for 30–60 s. Gene expression levels were calculated using the 2^−∆∆Ct^ method [52], with *elongation factor 1 alpha* (*EF1α*) and *ribosomal protein L28 (RPL28)* used as reference genes. RT-qPCR included three biological replicates, and each replicate had three technical replicates. Primer sequences are presented in Table 3.

### 4.8. Preparation of Double-Stranded RNA (dsRNA) and dsRNA/SPc Nanoparticle Complex

Double-stranded RNAs (dsRNAs) for the target genes *CYP405D1*, *CYP6AB269*, *CYP4AU1*, and the control ds*EGFP* were synthesized using the T7 RNAi Transcription Kit (Nanjing Vazyme Biotech Co., Ltd., Nanjing, China). PCR products of 328, 400, 364, and 413 bp for *CYP405D1*, *CYP6AB269*, and *CYP4AU1*, and the control ds*EGFP,* respectively, were amplified, and a T7 promoter sequence (TAATACGACTCACTATAGGG) was attached to the 5′ end of each primer to facilitate transcription. The PCR reaction mixture included 8 µL NTP Mix, 2 µL 10× transcription buffer, 2 µL T7 enzyme mix, and 8 µL DNA template, incubated at 37 °C for 2 h after mixing and centrifugation. Post-transcription, the dsRNA was purified using a double enzyme digestion system consisting of 20 µL transcription product, 17 µL RNase-free H₂O, 2 µL RNase T1, and 1 µL DNase I. Following purification, the quality and quantity of the dsRNA were measured using a Quawell UV-Vis Q5000 spectrophotometer (Quawell Technology Inc., San Jose, CA, USA).

The dsRNA for enhanced green fluorescent protein (ds*EGFP*) was used as a control. A star polycation (SPc) (obtained from China Agricultural University) was used to form the dsRNA/SPc nanoparticle complexes. The synthesized dsRNAs were mixed with SPc at a 1:1 mass ratio, with final concentrations of SPc and dsRNA at 500 ng/µL [28]. The mixture was incubated at room temperature for 15 min to allow the formation of dsRNA/SPc complexes. All samples were stored at −20 °C until further experimentation.

### 4.9. Nanocarrier-Mediated RNA Interference (RNAi) and Toxicity Bioassays

**Figure 8:**
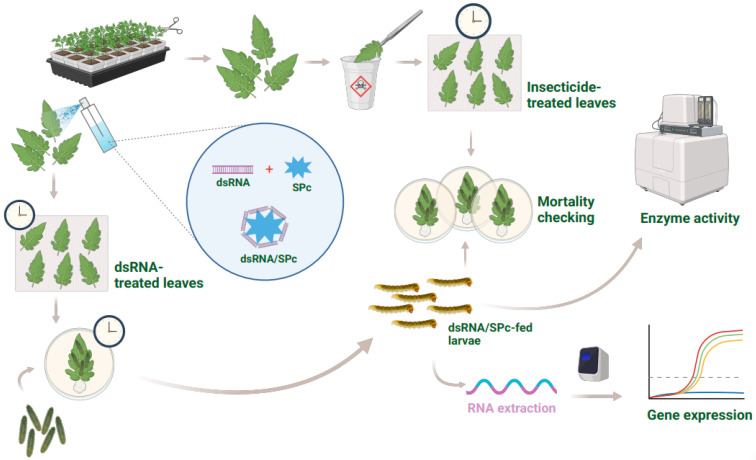
Schematic diagram of nanocarrier-mediated RNA interference.

The dsRNA/SPc nanoparticle complexes (ds*CYP405D1*/SPc, ds*CYP6AB269*/SPc, ds*CYP4AU1*/SPc, and ds*EGFP*/SPc) were sprayed onto tomato leaves at a concentration of 500 ng/µL (Figure 8). The leaves were dried at room temperature and placed in Petri dishes lined with filter paper. The petioles were covered with wet cotton wool to maintain moisture. Three replicates were conducted for each treatment, and each replicate included 20 *T. absoluta* larvae (2nd instar). Controls included ds*EGFP*/SPc and DEPC water. Experiments were conducted under laboratory conditions (25 ± 1 °C, 60 ± 5% relative humidity, 16:8 h light/dark photoperiod).

After 48 h of feeding on the treated leaves, surviving larvae were collected for RNA extraction and cDNA synthesis to assess gene silencing efficiency using RT-qPCR (Figure 1). Five larvae were pooled as one biological replicate. The susceptibility of the tetraniliprole-resistant strain (TetraRS) of *T. absoluta* to tetraniliprole was tested after 48 h of feeding on the dsRNA/nanoparticle complexes. This was conducted using the leaf-dip bioassay technique (described earlier) on tomato leaves treated with the LC_50_ of tetraniliprole for the TetraRS strain. Controls included DEPC water and ds*EGFP*/SPc. Insect mortality was recorded 48 h post-exposure. All bioassays were replicated three times and conducted under laboratory conditions.

### 4.10. Data Analysis

The bioassay data were analyzed using the log-probit model by POLO Plus 2.0 (LeOra Software, Petaluma, CA, USA). All data of *T. absoluta* related to life-history traits, enzyme activity, RT-qPCR, and mortality were analyzed by Student’s *t*-test and one-way analysis of variance (ANOVA) with Tukey’s HSD test to find significant differences. The statistical analysis was performed using SPSS version 29 (SPSS Inc., Chicago, IL, USA) with significance set at *p* < 0.05 for all treatments. Figures illustrating the life-history traits, enzyme activity, mortality, and expression levels of genes were generated using GraphPad Prism 9 (GraphPad Software, Waltham, MA, USA). The schematic figure was created with BioRender.com.

## Figures and Tables

**Table 1:** Selection of *Tuta absoluta* to tetraniliprole.

Generations	LC_50_ (95% CI) ^a^ mg/L	Slope ± SE ^b^	χ^2 c^	*p*-Value	RR ^d^
F0	0.03 (0.02–0.03)	2.37 ± 0.22	7.03	0.99	-
F1	0.03 (0.02–0.03)	2.22 ± 0.19	5.35	0.99	1.03
F2	0.04 (0.04–0.05)	2.05 ± 0.17	5.26	0.99	1.55
F3	0.08 (0.06–0.09)	1.80 ± 0.15	7.41	0.99	2.65
F4	0.15 (0.12–0.19)	1.53 ± 0.14	8.18	0.98	5.20
F5	0.25 (0.21–0.31)	1.94 ± 0.16	10.85	0.93	8.79
F6	0.36 (0.31–0.43)	2.14 ± 0.17	17.80	0.54	12.48
F7	0.48 (0.40–0.56)	2.20 ± 0.17	5.28	0.99	16.44
F8	0.60 (0.49–0.74)	1.70 ± 0.13	14.19	0.77	20.80

Number of larvae exposed in bioassay, including control = 480; df = 19. ^a^ 95% confidence interval. ^b^ Standard error. ^c^ Chi-square value (*χ*^2^) calculated by PoloPlus 2.0. ^d^ Resistance ratio (RR) = LC_50_ of resistant strain/LC_50_ of susceptible strain.

**Table 3:** The primer used for RT-qPCR and dsRNA synthesis.

Primer Name	Forward Sequence	Reverse Sequence
Tabs013762	TGCACGAGTTTCCAGGAGAT	TCTTCCCGTGTCTCTTCCAC
Tabs005394	GATCGTCCTAGCGCTTTGTG	GGCAGAGGTTTGTCGTGTTT
Tabs004410	TTCAACATGCACAGTACCGC	GGCTGTTTTCGGGAAGGAAG
Tabs007959	GCAGTCAAGGAAAGGCTAGC	TGTCGGTCACCTGTGTTTCT
Tabs009537	AGGACCAATCTGCCGAAAGA	AGTACAGCTGAAGGAACGCT
Tabs011636	ACTCTTACTCCGTGGCTGAC	CAGCGGTGTATTCGACCTTG
Tabs008791	AATCAGATCGACGCAGCAAC	CGGGTACAAGGCCGTAATTG
Tabs012471	GGATGGAGACTCGTTCGACT	GTACTTTCAACCGGCGGATC
Tabs011297	TTTCAAATCCTTCGGCCGTG	CCAGGATCGCTAGGGTTCTC
Tabs013496	GACAAAAGCGCGGGTAGTAG	CCACGGGTAGTCACATCCTT
*EF1α*	GAAGCCTGGTATGGTTGTCGT	GGGTGGGTTGTTCTTTGTG
*RPL28*	TCAGACGTGCTGAACACACA	GCCAGTCTTGGACAACCATT
*dsEGFP*	TAATACGACTCACTATAGGGAAGTTCAGCGTGTCCGGCGAGG	TAATACGACTCACTATAGGGCACCTTGATGCCGTTCTTCTGC
ds*CYP405D1*	taatacgactcactatagggGATACCGACCGTTCCAAAGA	taatacgactcactatagggAGAAAATTGTGGTTCGGTGC
ds*CYP6AB269*	taatacgactcactatagggACGAAAACTCCGCTTTCAGA	taatacgactcactatagggGCAAGCTGGTGTAACGTGAA
ds*CYP4AU1*	taatacgactcactatagggCATTGACTCCATCACCATCG	taatacgactcactatagggATCAGGGTTGAACAAGTCCG

## Data Availability

The authors confirm that the data supporting the findings of this study are available in the article.
